# The underactive bladder: detection and diagnosis

**DOI:** 10.12688/f1000research.7344.1

**Published:** 2016-01-25

**Authors:** Nadir Osman, Altaf Mangera, Christopher Hillary, Richard Inman, Christopher Chapple

**Affiliations:** 1 Department of Urology, Sheffield Teaching Hospitals NHS Trust, Sheffield, UK

**Keywords:** Underactive bladder, detrusor underactivity, chronic urinary retention

## Abstract

The inability to generate a voiding contraction sufficient to allow efficient bladder emptying within a reasonable time frame is a common problem seen in urological practice. Typically, the symptoms that arise are voiding symptoms, such as weak and slow urinary flow. These symptoms can cause considerable bother to patients and impact upon quality of life. The urodynamic finding of inadequate detrusor contraction has been termed detrusor underactivity (DUA). Although a definition is available for this entity, there are no widely accepted diagnostic criteria. Drawing parallels to detrusor overactivity and the overactive bladder, the symptoms arising from DUA have been referred to as the “underactive bladder” (UAB), while attempts to crystallize the definition of UAB are now ongoing. In this article, we review the contemporary literature pertaining to the epidemiology and etiopathogenesis of DUA as well as discuss the definitional aspects that are currently under consideration.

## Introduction

Detrusor underactivity (DUA) is a common bladder dysfunction observed in both men and women undergoing urodynamic studies for the assessment of lower urinary tract symptoms (LUTS). It is typically associated with voiding LUTS such as slow urinary flow and intermittency but may also result in storage and post-micturition LUTS. Despite being commonly encountered in routine clinical practice, DUA has been given relatively little attention in clinical and scientific research. Not only can DUA arise in bothersome symptoms but its occurrence may impact upon the outcome of treatments for other dysfunctions, such as bladder outlet obstruction and stress urinary incontinence. Recently, there has been renewed interest in DUA, the associated symptoms, and potential treatments. This article aims to review the recent literature regarding the epidemiology, etiopathogenesis, and diagnosis of DUA and its associated symptoms.

## Definition

A plethora of terms have been used to describe a bladder that does not contract efficiently. Terms such as detrusor areflexia, hypotonic bladder, atonic bladder, detrusor failure, impaired detrusor contractility, and chronic retention all reflect the lack of consensus and uncertainty as to the pathophysiological mechanisms at play. In an effort to standardize terminology, the International Continence Society report in 2002 used the term DUA to describe a urodynamic abnormality. This was defined as “a contraction of reduced strength and/or duration, resulting in prolonged bladder emptying and/or failure to achieve complete bladder emptying within a normal time span” ^1^. This definition is necessarily vague on specifying the parameters for length and strength of contraction and prolonged voiding, as what is normal has not yet been established.

## Signs and symptoms

There is a lack of prospective studies correlating DUA and its associated clinical signs and symptoms. In clinical practice, the typical presentation is of voiding LUTS such as reduced flow, prolonged flow, hesitancy, and intermittency. After voiding, some patients may report a sensation of incomplete bladder emptying, which may be related to a raised post-voiding residual (PVR).

One could speculate that the presence of storage symptoms is related to the bladder sensory function in the individual patient, such that patients with poor or absent bladder sensation will have a reduced urge to pass urine, which will result in infrequent voiding. Anecdotally, some patients may report they lack any perception of their bladder emptying during the voiding process. Should these patients retain a large enough PVR, they may also experience incontinence, particularly during the night. This clinical picture is commonly termed chronic urinary retention (CUR). Patients with retained bladder sensation may void with a normal frequency pattern or have increased frequency, nocturia, and incontinence should the PVR be high enough.

There has been some debate about whether a symptom complex is associated with DUA, referred to by some as the underactive bladder (UAB), which is rather analogous to detrusor overactivity (DO) and the overactive bladder (OAB) symptom complex. This is certainly an attractive concept, as it could, in theory, allow patients to be diagnosed and treated on the basis of symptomatology. Nevertheless, there are clear difficulties in defining a specific set of symptoms that do not overlap significantly with LUTS due to benign prostatic enlargement (BPE). Clearly in women this is less likely to be problematic given the relatively rare occurrence of obstructive causes. A working definition for UAB was recently proposed by an expert consensus group: “…a symptom complex suggestive of detrusor underactivity and is usually characterised by prolonged urination time with or without a sensation of incomplete bladder emptying, usually with hesitancy, reduced sensation on filling, and a slow stream” ^2^.

## Epidemiology

Given the inability to diagnose DUA without an invasive urodynamic study, it is unsurprising that there is little information on its prevalence on a population basis. There is no single non-invasive proxy measure (e.g. raised PVR, reduced urinary flow rate) that could reliably identify patients with the condition. Clinical series of patients with LUTS undergoing urodynamic studies offer the best available evidence as to the prevalence of DUA. In younger men (<50 years), prevalence is 9–28%, which rises to 48% in elderly men (>70 years) ^3^. In elderly women, prevalence rates are between 12 and 45% and peak in the institutionalized elderly. Older frail patients often have co-existent DO, and this entity was originally described by Resnick and co-workers in 1987 as detrusor hyperactivity impaired contractility (DHIC) ^4^.

## Etiopathogenesis

Given the diverse patient groups who can be affected by DUA, it is clear that the etiopathogenesis is likely to be multi-factorial. There seems to be an assumption in the literature that aging leads to a decline in detrusor contractile function. While plausible, it must be emphasized that there is no conclusive evidence that this is the case. The available data from animal and human studies is often quite contradictory.

The etiological factors resulting in DUA can be broadly classified as neurogenic or myogenic.

### Myogenic factors

Any pathological process that changes the normal structure and/or functioning of the extracellular matrix or the myocytes of the detrusor may cause a loss in the generation or transmission of a contraction. Factors that may affect the contractile activity of the myocytes include disruption of important cellular mechanisms (e.g. ion storage/exchange, excitation-contraction coupling, calcium storage, and energy generation). The implication of this is that even if efferent neuronal activity is normal, an impaired contraction will result ^5^.

Supporting the idea that myogenic factors may be important, morphological changes have been observed within both the normally aged and the diseased detrusor. Much of this work was conducted by Elbadawi, who suggested that specific ultrastructural features detected by electron microscopy were associated with aging and different bladder dysfunctions including DUA ^6– 8^. The specific pattern associated with DUA was described as the “degeneration” pattern, characterized by widespread disrupted myocytes with axonal degeneration ^6^. What is not clear is whether detrusor myocyte disruption is in itself the cause of DUA or the result of an underlying pathological process.

### Neurogenic factors

Intact bladder sensation is central to the voiding reflex. The afferent nerves monitor bladder volume during the storage phase and also during the voiding phase. Afferent nerves arising at the level of the urethra may also have a contributory role in perceiving flow through the urethra ^9, 10^. If afferent functioning is reduced, this may lead to a reduction in the strength or premature termination of the voiding reflex ^11^.

### Bladder outlet obstruction

It has long been held that DUA occurs as a secondary effect of prolonged bladder outlet obstruction (BOO). This assumption has been largely derived from work in animals, particularly the rodent, where partial BOO is induced by a constricting metal ring or ligature. The process by which DUA occurs has been widely described and includes three phases ^12, 13^:

1. Following obstruction, there is increased bladder outlet resistance leading to bladder wall distension
2. Compensatory hypertrophy and hyperplasia of the detrusor then occurs associated with a rise in tissue blood supply
3. Initially, contractile function is maintained; however, after a variable period of time, contractility is dissipated with a reduction in bladder emptying ability (decompensation)

Relating the insights from this work to humans is problematic, as the animals studied are usually young and female with an acute obstruction induced using a constricting ring. This is unlikely to be a good representation of the common clinical scenario of an elderly man with compressive benign prostatic obstruction. Interestingly, the largest longitudinal study available has shown that in man, prolonged BOO does not usually result in decompensation and DUA. In a group of 170 men with BOO demonstrated on pressure-flow studies, no significant deterioration in urodynamic parameters was observed at a mean follow up of 13.9 years (no change in pdet@Qmax [detrusor pressure at maximum flow] and a reduction in Qmax [maximum flow rate] of only 1 ml/s) ^14^.

### Diabetes mellitus

Diabetic bladder dysfunction (DBD) (or diabetic cystopathy) is a well-recognized cause of DUA. Diabetes mellitus probably affects function through both a myogenic and a neurogenic mechanism leading to a decline in bladder emptying ability throughout the course of the disease ^15, 16^. It is traditionally thought that this predominately occurs through hyperglycemia-induced axonal degeneration and segmental demyelination leading to impaired afferent and efferent bladder function (autonomic neuropathy) ^17^. Myogenic mechanisms are less clearly elucidated, though it is likely that changes in intercellular connections and excitability, intracellular signaling, and receptor density and distribution are implicated ^18^.

## Diagnostic aspects

Currently, the only accepted modality for diagnosing DUA is an invasive pressure-flow test (or urodynamic study). However, there are no widely accepted criteria for diagnosing DUA. Most proposed methods relate to the measurement of the strength of the detrusor contraction.

### Detrusor contraction strength

A detrusor contraction generates pressure and flow ^19^; therefore, many authors have used two measurements to approximate contraction strength and thereby diagnose DUA: the maximal flow (Qmax) and the detrusor pressure at maximal flow (Pdet@Qmax). Most often a reduction in either is considered to be lower than the “normal” range. Historically, for men the normal ranges are derived from a series of men undergoing de-obstructive surgery ^20, 21^. In other groups, such as healthy men and women, in particular, the ranges are less well established ^22– 24^. Although relatively simple to apply, this method has two flaws. Firstly, it is likely to be an inaccurate method of determining the maximal pressure generated by the bladder due to the bladder outlet relation (BOR), the normal inverse correlation between detrusor pressure and urine flow during voiding ^25^. Essentially, the BOR can be summed up as during voiding the pressure is highest when flow is lowest and vice versa, hence the pdet@Qmax represents the point of lowest bladder pressure during voiding. Secondly, the fact that the flow rate is also impacted upon by the degree of bladder outlet resistance is not considered. This is important because when detrusor pressure is low, the cause of low flow may also have its basis in obstruction (e.g. due to BPE) or alternatively a normal Qmax can result from reduced outlet resistance even if detrusor pressure is low (e.g. post-prostatectomy incontinence).

In order to gain a more accurate assessment of contraction strength, different methods of measuring isovolumetric bladder pressure have been introduced:

1. Watts factor

This is an estimate of the power per unit area of the bladder surface that is generated by the detrusor. The formula for calculation is *WF = [(pdet + a) (vdet + b) – ab]/2π* where vdet is detrusor shortening velocity and a and b are fixed constants (a=25 cmH _2_O, b=6 mm/s) derived from experimental and clinical data ^26^. The main pros of the WF are that it is not dependent on bladder volume ^26^ and is not influenced by increased outlet resistance ^27^. There are, however, no validated values for what is considered normal. Currently, the WF is little used in practice due to its complexity as well as the fact that it assesses only strength of contraction, rather than duration.

2. Projected isovolumetric pressure (PIP)

The PIP was proposed by Schäfer, who used a straight line to represent the BOR and then “projected” back to the y-axis (pdet) from the point representing pdet@Qmax to obtain the isovolumetric pressure. This projection is calculated by the formula *PIP = Pdet@Qmax + KQmax* where K is a fixed constant representing the steepness of the angle of the BOR to x-a axis ^28^. K is dependent on the specific population studied and differs between men and women ^29^; in men it is usually taken as 5. The suggested groupings for PIP are as follows:

-     >150 − strong contractility
-     100 to 150 − normal contractility
-     50 to 100 − weak contractility
-     <50 − very weak contractility

There are two other reported variants of the PIP: the detrusor coefficient (DECO), which is the PIP divided by 100, and the bladder contractility index (BCI), which is essentially the same as PIP but with different groupings. The main advantage with these formulae is that they are easy and quick to use; however, they may overestimate PIP and have less test-retest reliability than measuring isovolumetric pressures directly ^30^.

3. Mechanical occlusion of flow

This entails the direct measurement of isovolumetric pressure by the mechanical obstruction of urine flow ^31^. There are two methods for this measurement: (1) a stop test, the interruption of flow once it has already started, or (2) continuous occlusion test, where urine flow is impeded before and throughout the duration of the detrusor contraction. Stop tests can be voluntarily induced by the patient by contraction of the rhabdosphincter or alternatively the urodynamicist can impede flow through methods such as manual occlusion of the urethra. The voluntary stop test tends to underestimate pressure by about 20 cmH _2_O, which is likely to be attributable to a reflex detrusor inhibition brought on by sphincter contraction ^30, 32^. The voluntary test may not be possible in patients with sphincter weakness and in the elderly, whereas continuous occlusion can be painful and does not provide the opportunity for synchronous flow measurement.

## Detrusor contraction speed

If a bladder is slow to contract, then this could also potentially result in DUA. Cucchi and co-workers proposed the parameter of detrusor shortening velocity, as calculated by the formula vdet = Q/2[3/(V + Vt)/4π] ^0.66^ where Q represents the flow rate (ml/s), V represents bladder volume (ml), and Vt represents the volume of non-contracting bladder wall tissue. They found that a reduction in shortening velocity was evident before a decline in Watt factor in both men ^33, 34^ and women ^35^.

## Ambulatory urodynamics

It is not uncommon for a patient to be unable to void during a urodynamic study due to anxiety; this has been termed “bashful bladder”, which has been attributed to inadequate relaxation of the rhabdosphincter combined with reflex detrusor inhibition. This situation is usually identified by taking a good clinical history of previous voiding symptoms; however, if doubt persists, then an ambulatory urodynamic study may be helpful ^36, 37^.

## Conclusions

DUA remains a poorly characterized and poorly understood bladder dysfunction. Its clinical correlate, UAB, has recently been introduced into clinical usage, yet it remains unclearly defined. To progress the current situation, there is a need to investigate the common mechanisms by which patients develop DUA as well as to collect prospective data that can be used to correlate the symptoms and signs with an underlying urodynamic abnormality.
