# Supplementary material for: The underactive bladder: detection and diagnosis
Source: F1000Res. 2016 Jan 25;5:F1000 Faculty Rev-102. [Version 1] doi: 10.12688/f1000research.7344.1 (PMC4815614; doi:10.12688/f1000research.7344.1)
Supplement: Peer review history

# Peer review: Referee response for version 1

**DOI:** 10.5256/f1000research.7915.r12061

**Published:** 2016-01-25

**Authors:** Peter F. W. Rosier

**Affiliations:** 1 Department of Urology, University Medical Center Utrecht, Utrecht, Netherlands

The short commentary ‘The underactive bladder: detection and diagnosis’ discusses the topic from the perspective of symptoms, epidemiology, pathogenesis, and diagnostic aspects. The paragraphs on diagnostic aspects are a summary of urodynamic testing, more specifically: pressure-flow analysis. I assume that the words ‘…are typically associated with…’ in the introduction and the words ‘…typical presentation…’ in the signs and symptoms section, refer to the historical beliefs of professionals. The last paragraph of the signs and symptoms section mentions that there is a debate on whether there is a symptom complex associated with detrusor underactivity (the underactive bladder syndrome?). I agree with this statement and careful reading of the working definition cited from reference 2 in the commentary makes it obvious that the working definition of (the) bladder outflow obstruction (syndrome) would be exactly identical, if it was introduced nowadays. The elements of the working definition for UAB (as a signs and symptoms syndrome) are equal to those considered, both currently and traditionally, very relevant in men with benign prostatic enlargement ^1^. I conclude that the underactive bladder syndrome as it is suggested here is not specific, like all LUTD-syndromes, and that, indeed, underactive detrusor (contraction during voiding) is a condition only assessable with a pressure-flow study.

There is some observational clinical data on how the detrusor compensates for the growing prostate and the–by consequence–increase in bladder outflow obstruction, in addition to the animal studies referred to in the commentary, to explain the pathophysiology ^2^. With the perspective of DU, this clinical study may allow two interpretations; first: the detrusor of aging men increases (maximum) power to compensate for the growing prostate and second: some detrusors are better able to compensate than others. The validity of the clinical observation ^2^ was subsequently confirmed in a canine experiment ^3^. If relevant neurogenic abnormalities are excluded on the basis of history and clinical examination, myogenic factors remain as the cause of detrusor underactivity, and based on biologic variance, some detrusors will be stronger than others and some will be better able to conquer challenges. The search for relevant amino-acids or other molecules will be important here if we want to discover ways to improve the symptomatically weak detrusors.

Urodynamic diagnosis of detrusor contraction work or power during voiding is not yet standardized, and the commentary gives a neat overview of parameters that may prove to be useful. Especially objective parameters that will demonstrate predictive value and responsiveness to management will become relevant in the future.

Cooperation with the patient is necessary to obtain a representative pressure-flow test, and the autonomic voiding reflex requires parasympathetic dominance in a–for the patient involved–stressful situation. This is even more important when the patient has a ‘bashful bladder’. I consider it valuable that this is introduced here in this context. Based on the relevance of this dysfunction, that will exist in a continuum from ‘low grade’ to ‘very troublesome‘, the new ICS Good Urodynamic Practices and Terms (that will be available in 2016) will introduce the terms (or diagnoses) ‘situational inability to void’ and ‘situational inability to void as usual’. Whether ambulatory urodynamics provides a reliable method to obtain a pressure-flow test remains speculative however, because of the lack of evidence.

I have read this submission. I believe that I have an appropriate level of expertise to confirm that it is of an acceptable scientific standard.

## References

[1] AbramsPChappleCKhourySRoehrbornCde la RosetteJ: Evaluation and treatment of lower urinary tract symptoms in older men. *J Urol*.2009;181(4) : 10.1016/j.juro.2008.11.127 1779-87 10.1016/j.juro.2008.11.127 19233402

[2] RosierPFde WildtMJde la RosetteJJDebruyneFMWijkstraH: Analysis of maximum detrusor contraction power in relation to bladder emptying in patients with lower urinary tract symptoms and benign prostatic enlargement. *J Urol*.1995;154(6) :2137-42 7500477

[3] LecamwasamHSSullivanMPCravalhoEGYallaSV: Detrusor internal and external work in relation to passive urethral resistance in a canine model of the lower urinary tract. *Neurourol Urodyn*.1996;15(5) : 10.1002/(SICI)1520-6777(1996)15:5<529::AID-NAU9>3.0.CO;2-H 529-43; discussion 544 10.1002/(SICI)1520-6777(1996)15:5<529::AID-NAU9>3.0.CO;2-H 8857620

# Peer review: Referee response for version 1

**DOI:** 10.5256/f1000research.7915.r12060

**Published:** 2016-01-25

**Authors:** Naoki Yoshimura

**Affiliations:** 1 Department of Urology, University of Pittsburgh, Pittsburgh, PA, USA

I have read this submission. I believe that I have an appropriate level of expertise to confirm that it is of an acceptable scientific standard.
